# Monthly Summary

**Published:** 1875-11

**Authors:** 


### MONTHLY SUMMARY.

---

*Adulteration of Beeswax.*—For a year or two past there has been offered in this market, and most probably elsewhere, an article termed "refined beeswax." It is unusually handsome in appearance and is generally represented as being strictly pure. It may be known by all of it being of a uniform bright-yellow color, entirely free from the sedimentary stratum of impurities ordinarily found in country wax. Its surface is clean and glossy, having no foreign particles adhering to it. On account of these apparent merits, it is usually sold at an advance on the price of the regular article. All of this so-called refined beeswax, so far met with, has been moulded into the shape of oblong blocks of uniform size, measuring about fourteen inches in length, eight inches in width and three in thickness, tapering slightly upwards, and weighing about eight pounds on an average.

The melting point of the refined wax was found to be 146° F., that of pure wax being 156° and that of paraffin from 137° to 140°. Its specific gravity is .929, placing it again intermediate between beeswax, .963, and paraffin, .871. Being thus induced to suspect the presence of the latter body, 100 grains of the refined article were heated for fifteen or twenty minutes with one ounce of sulphuric acid to about 350° F.; several ounces of water were then added; and after cooling a sheet of paraffin weighing 80 grains was obtained, the loss representing the beeswax which had been carbonized by the acid. In order to verify the experiment, it was repeated with a composition of four parts paraffin to one of wax, when analogous results were obtained. 100 grains of pure paraffin, treated in the same manner, were recovered unchanged.

All the best text-books recommend fuming Nordhausen acid for this purpose, and state that an allowance must be made for a portion of paraffin charred by this acid. No one seems to have previously tried the ordinary commercial sulphuric acid, which was really found to be better adapted than the Nordhausen, as all the wax was carbonized and none of the paraffin affected. The test is very readily applied, the only difficulty, and this a very trivial one, being the separation of the carbonaceous matter from the paraffin. It is most conveniently removed by repeatedly melting the paraffin on water, at the same time gently stirring it, so that the black particles can subside.

There seems to be a considerable difference in the mode of contraction, while cooling, between beeswax and paraffin, and this may serve to detect the adulteration, at least when practiced to this extent. Blocks of paraffin are decidedly concave on the top, and the specimens of adulterated wax presented herewith will be observed to be more or less concave on top in proportion to the amount of paraffin which they contain. Pure beeswax appears to be level, and the contraction acting in a horizontal direction and tending rather to the production of vertical fissures.

The optical behaviour is also different; pure wax is quite opaque, while this adulterated article is somewhat translucent, more particularly on the edges.

Although no injury is likely to result from this admixture, it is an evident fraud, as there is considerable difference in the commercial value of the two substances. It may be added, in this connection, whether it is not time for the Pharmacopœia Committee to turn their attention to paraffin, since we have thus again detected it forcing its way into pharmacy under the garb of beeswax, cosmolin, and vaselin.—*Druggists' Circular.*

---

*A Photographic Wonder.*—Dr. Ultzmann, teacher at the University of Vienna, lately read a paper before the Medical Society of Lower Austria, on the "Use of Photography in Medical Studies." *Inter alia*, he mentioned, on the authority of Dr. Vogel, that an eruption of small-pox had been made evident by photography twenty-four hours before it actually came out. Although no one could as yet observe anything on the skin of the patient, the negative plate showed stains on the face which perfectly resembled the variolous enan-them, and twenty-four hours afterward the eruptiou became clearly evident —*Med. and Surg. Journal.*

*External Use of Chloral Hydrate.*—A writer in the *Canada Medical Record* recommends a solution of Chloral Hydrate in water, from 3 to 5 grains to the ounce, as a substitute for carbolic acid for external use. Its effect on ulcers is prompt and admirable, and a case of eczema was successfully treated by it. In cases of amputation, where the surfaces took on unhealthy action, the solution changed their character and removed the difficulty in twenty-four hours. It removes fetor, is clean and neat, does not stain, and stimulates granulations without destroying them.

Chloral hydrate has also been used in Germany as an antiseptic application in cancer of the uterus. A strong solution—one part to twelve of water—is applied by means of cotton. Dr. Goodell of Philadelphia also speaks highly of it as a purifying and deodorizing agent.—*Pacific Med. and Surg. Journal.*

---

*The Anti-Neuralgic Properties of the Essence of Mint.*—In a paper read before the Societe de Therap, M. Delioux de Savinac points out that the essence of mint exerts a special influence on the sensory nerves, which diminishes the abnormal vivacity of their reactions when these become exalted into painful affections. It is comparable in this respect to chloroform, ether, and camphor. Though its high price will prevent its being extensively used in the pure state as an anti-neuralgic and antirheumatic, it may be employed in alcoholic solution as an application in the form of liniment, and camphor, chloroform, and laudanum may be added to it with advantage in various cases. Dr. Savignac has found it extremely useful in relieving the pain of pruritus; in alleviating, when employed in the form of injections, the miseries of vulvo-vaginal hyperæsthesias, vaginismus, and neuralgias of the head and face, which are best treated by making a small ball of wool of the size of a nut which has been made to imbibe a few drops of the essence, and rubbing the painful part with it gently. It is then to be covered with a larger piece of wool, and the whole kept in position for a few minutes with the palm of the hand. The success of these external applications of the essence of mint in neuralgias of the head and face, and even in cases of congestive cephalalgia, has been both prompt and permanent. He adds, however, that the cause of the pain must be local for the cure to be permânent, since, if it be dependent on hysteria, or be connected with intermittent fever, or with gastric derangement, the primary cause must first be removed. The remedy seems to have been nlog known in China. For intermuscular and parenchymatous neuralgias, which are especially obstinate, he has tried the following as a hypodermic injection:

Hydrochlorate of morphia, 1½ grains.
Mint water, 140 minims.
Alcoholate of mint, 15 minims. Mix.

